# Structural abnormalities in the placenta

**DOI:** 10.1186/1471-2393-12-S1-A3

**Published:** 2012-08-28

**Authors:** Harvey J Kliman

**Affiliations:** 1 Yale University School of Medicine, New Haven, CT, USA

## 

Genes regulate the development of fertilised egg into the inner cell mass (which will become the embryo, fetus and, eventually, baby) and the trophoblast (which will become the placenta). Defects in the genes that regulate these processes lead to a wide range of embryonic, fetal and neonatal defects, from minor cosmetic abnormalities, to such severe defects disasters that a pregnancy miscarries within a few days to weeks after fertilization. Since the placenta and fetus share the same genome, genetic defects in the fetus are often mirrored in the placenta as abnormal growth patterns. Abnormal growth pattern of the trophoblast layers, namely trophoblast invaginations, appear to be associated with genetic defects in the fetus. When these deep pits are cut in cross section they appear as trophoblast inclusions. Compared to the placentas from normal children there is a significantly increased frequency of trophoblast inclusions in cases of known chromosomal diseases, such as trisomy 21, 13 and 18, as well as triploidy.

